# ARHGEF11 promotes proliferation and epithelial-mesenchymal transition of hepatocellular carcinoma through activation of β-catenin pathway

**DOI:** 10.18632/aging.103772

**Published:** 2020-10-29

**Authors:** Jinpeng Du, Zexin Zhu, Lin Xu, Xing Chen, Xuefeng Li, Tian Lan, Wei Li, Kefei Yuan, Yong Zeng

**Affiliations:** 1 Department of Liver Surgery and Liver Transplantation Center, West China Hospital, Sichuan University, Chengdu 610041, China; 2 Laboratory of Liver Surgery, West China Hospital, Sichuan University, Chengdu 610041, China; 3 School of Basic Medical Sciences, Guangzhou Medical University, Guangzhou 511436, P. R. China; 4 Shenzhen Luohu People’s Hospital, The Third Affiliated Hospital of Shenzhen University, Shenzhen 518001, P. R. China

**Keywords:** hepatocellular carcinoma (HCC), epithelial-mesenchymal transition (EMT), β-catenin, prognosis, biomarker

## Abstract

Rho guanine nucleotide exchange factor 11 (ARHGEF11) has been proved to promote tumor metastasis in glioblastoma and ovarian carcinoma. However, the role of ARHGEF11 in hepatocellular carcinoma (HCC) progression is largely unknown. Here, we found that ARHGEF11 was upregulated in HCC samples and highly metastatic hepatoma cell lines. Knockdown of ARHGEFF11 inhibited the cell proliferation and invasion in both HCCLM3 and SKHEP1 cell lines. Subsequent mechanistic investigation showed that downregulation of ARHGEF11 significantly attenuated β-catenin nuclear translocation, thereafter repressed the expression of ZEB1 and cyclinD1, finally contributing to inhibition of epithelial-mesenchymal transition (EMT) and cell cycle arrest. Moreover, high levels of ARHGEF11 were found to be associated with shorter disease free and overall survival. A prognostic nomogram model that integrated ARHGEF11, tumor size and BCLC classification showed good performance in predicting clinical outcomes of HCC patients. Overall, this study demonstrated that ARHGEF11 could promote proliferation and metastasis of HCC via activating β-catenin pathway, suggesting that ARHGEF11 might serve as a potential prognostic biomarker for HCC.

## INTRODUCTION

Hepatocellular carcinoma (HCC) has been ranked as the sixth most common human malignancies worldwide, with 782000 cases diagnosed in 2012 [1]. Despite great advances in surgical procedures and chemical therapies over the past decades, the overall prognosis of HCC patients is still poor, with a 5-year survival of only 18% [2]. Although various molecular events or biological processes, including somatic DNA alterations, abnormal expression of oncogenes and aberrant activations of tumorigenic pathways, have been considered to induce hepatocarcinogenesis [3–5], limited effective interventions can be used in clinical management. Therefore, it is urgent that we explore the underlying molecular mechanisms of the development and progression of HCC for developing alternative therapeutic strategies of HCC.

Epithelial-mesenchymal transition (EMT), a biological process of conversion of epithelial cells to mesenchymal cells, involves the loss of cell-cell adhesion and acquisition of migratory and invasive phenotype [6]. EMT has been recognized as an important element in HCC progression [7, 8]. Loss of E-cadherin is an important event during EMT. A series of signaling pathways have been proved to regulate E-cadherin expression, such as Wnt/β-catenin signaling. Under pro-malignant conditions, activated Wnt pathway enforces β-catenin to disassociate from E-cadherin and translocate into the nucleus to activate the expression of ZEB1, a transcription repressor of E-cadherin [9]. However, the molecular mechanisms that regulate E-cadherin/β-catenin interaction are largely unknown.

Rho guanine nucleotide exchange factor 11 (ARHGEF11), which is also known as PDZ-RhoGEF or GTRAP48, is a Rho guanine nucleotide exchange factor (RhoGEF). RhoGEFs can selectively activate the RhoA family cytosolic GTPase, which can regulate cellular functions including cell cycle progression, cell migration, neurite growth, cell-cell adhesion and others [10, 11]. Recent studies have demonstrated that ARHGEF11 could induce metastasis in glioblastoma [12] and ovarian carcinoma [13] by promoting cell invasion and migration. Moreover, the exon 38-containing ARHGEF11 splice isoform was closely associated with the malignant phenotype of breast cancer [14]. However, the impact of ARHGEF11 on HCC has yet to be determined.

In the current study, it was shown that upregulation of ARHGEF11 could promote the proliferation, migration and invasion of hepatoma cells through induction of β-catenin nuclear translocation. In addition, we found that high levels of ARHGEF11 were related to poor prognosis of HCC patients. The prognostic nomogram model containing ARHGEF11 displayed good discrimination performance, which indicated that ARHGEF11 might be a potential biomarker of HCC.

## RESULTS

### ARHGEF11 is elevated in HCC patients

**Figure 1:**
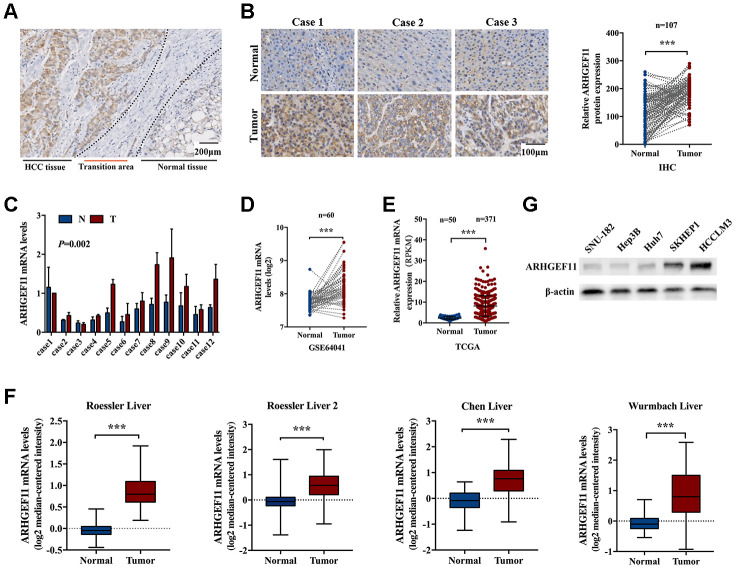
**ARHGEF11 is upregulated in HCC tissues.** (**A**) Representative IHC staining of ARHGEF11 in HCC tissues, scale bar 200 μm. (**B**) Three representative IHC staining images of ARHGEF11 in HCC and para-tumor tissues (left), scale bar 100 μm. Quantification of ARHGEF11 expression of paired HCC samples (n=107, right). Data are shown as mean ± SD. (**C**) Representative qRT-PCR results of ARHGEF11 expression of human HCC samples. Data are shown as mean ± SD. (**D**–**F**) Relative ARHGEF11 mRNA expression in GEO dataset (GSE64041), TCGA dataset and four Oncomine datasets respectively. (**G**) Relative ARHGEF11 expression in five hepatoma cell lines, as detected by western blot. ***P<0.001.

To identify the potential role of ARHGEF11 in HCC, the expression of ARHGEF11 in HCC tissues and paired noncancerous liver tissues was assessed via IHC staining. The IHC staining results demonstrated ARHGEF11 expression in HCC tissues were obviously upregulated compared with the adjacent normal liver tissues (Figure 1A and 1B). To validate the IHC results, the mRNA levels of ARHGEF11 in 12 paired human HCC samples were determined using qRT-PCR analysis. In spite of interindividual variations, the transcription levels of ARHGEF11 were significantly higher in HCC samples than that of matched noncancerous tissues (Figure 1C). Furthermore, public HCC datasets were used to compare ARHGEF11 expressions between tumor and normal tissues. The gene expression based on Gene Expression Omnibus dataset (GEO, GSE64041) (Figure 1D), The Cancer Genome Atlas (TCGA) dataset (Figure 1E) and four Oncomine datasets (Figure 1F) all showed similar results: ARHGEF11 was upregulated in HCC tissues. In additon, five hepatoma cell lines were used to detected ARHGEF11 expression levels. Intriguingly, HCCLM3 and SKHEP1 cell lines (highly metastatic) showed higher ARHGEF11 levels compared with SNU-182, Hep3B and Huh7 cell lines (weakly metastatic) (Figure 1G). Collectively, the aforementioned results suggested that ARHGEF11 might promote HCC progression.

### Upregulation of ARHGEF11 enhances the proliferation and migration of hepatoma cells

**Figure 2:**
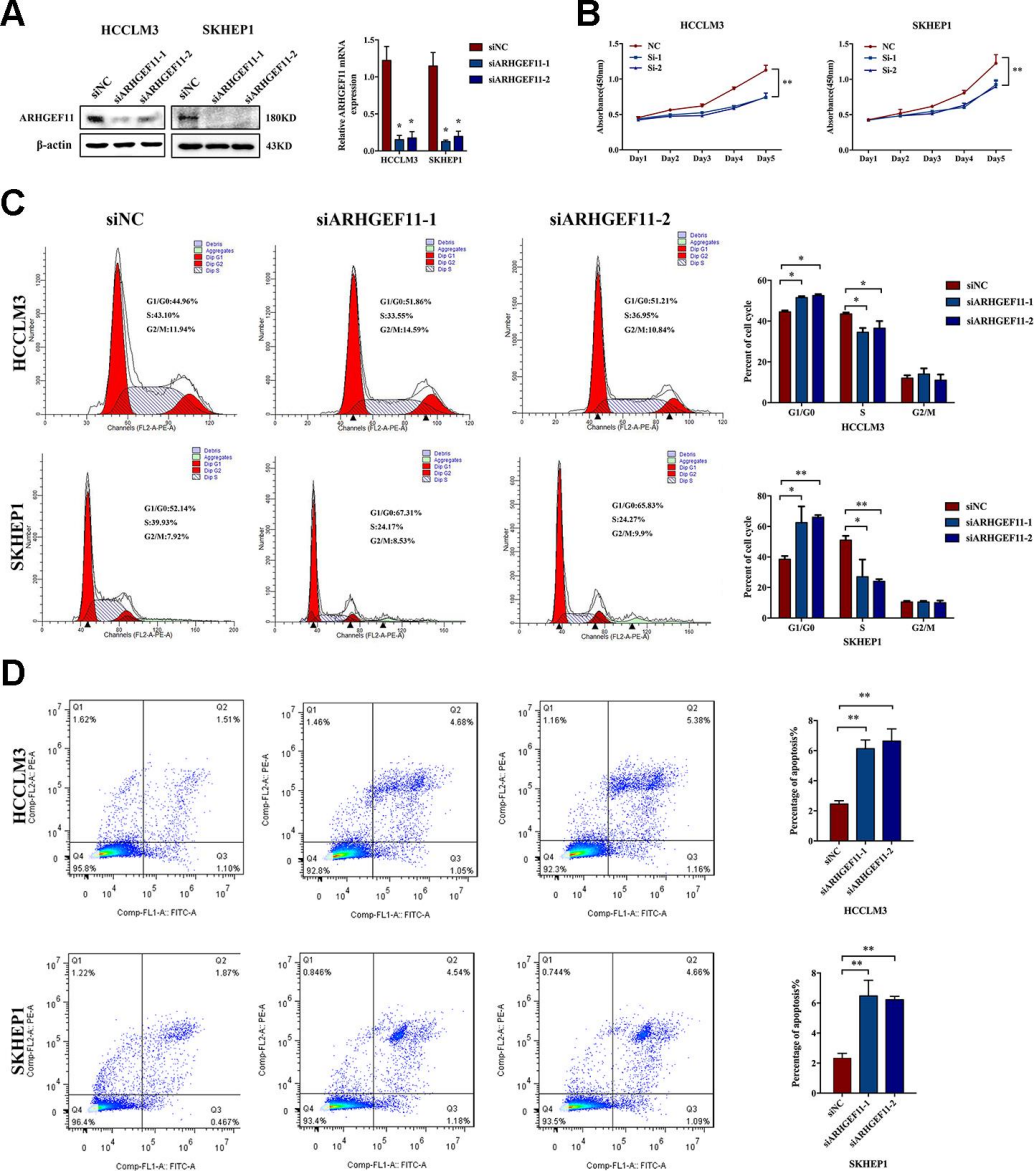
**Knockdown of ARHGEF11 induces cell growth inhibition, cell cycle arrest and cell apoptosis in hepatoma cell lines.** (**A**) ARHGEF11 knockdown with two different siRNAs (siARHGEF11-1, siARHGEF11-1) or negative control (siNC) as examined by western blot and qRT-PCR. (**B**) The viability of hepatoma cells detected by CCK8 assay. (**C**) Cell cycle distribution detected by flow cytometry. The representative images (left) and percentage of cell cycle (right) are shown. (**D**) Cell apoptosis detected by flow cytometry. The representative images (left) and relative percentage of apoptotic cells (right) are shown. Data are shown as mean ± SD. *P<0.05, **P<0.01.

**Figure 3:**
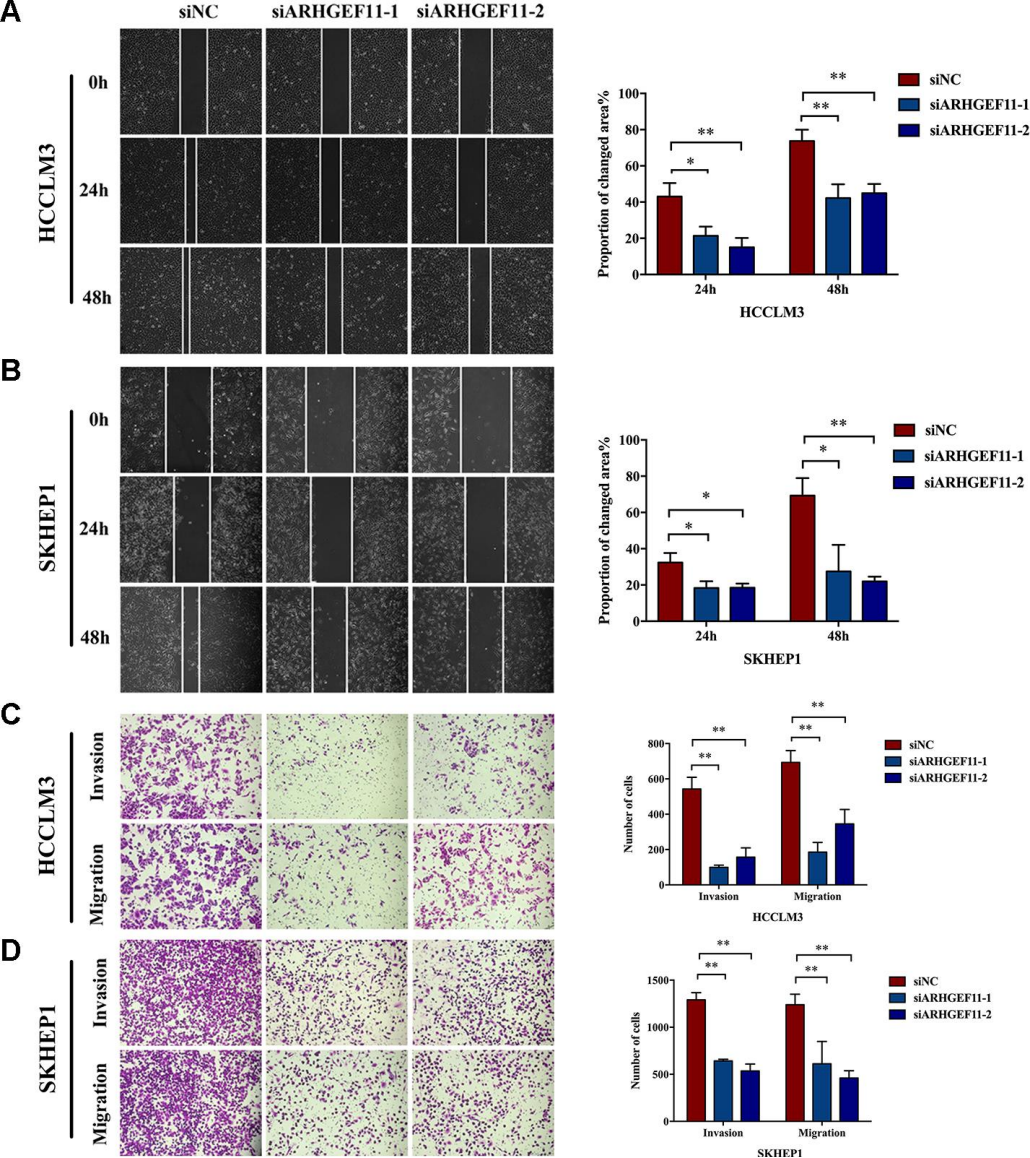
**Knockdown of ARHGEF11 suppresses the migration and invasion of hepatoma cells.** (**A** and **B**) Migration ability examined by Wound healing assay. The representative images (left) and proportion of changed area (right) are shown. (**C** and **D**) The migration and matrigel invasion ability detected by Transwell assay. The representative images (left) and average cell number on the bottom membrane (right) are shown. Data are presented as mean ± SD. *P<0.05, **P<0.01.

To understand the functions of ARHGEF11 upregulation in HCC progression, we silenced ARHGEF11 expression with two siRNAs (siARHGEF11-1 and siARHGEF11-2) in two highly metastatic hepatoma cell lines: HCCLM3 and SKHEP1. Successful siRNA-mediated knockdown of ARHGEF11 in both cell lines was confirmed via western blot and qRT-PCR analysis (Figure 2A). ARHGEF11 downregulation significantly inhibited the cell viability of hepatoma cells as detected by CCK-8 assay (Figure 2B). To further explore the ARHGEF11 roles in proliferation of hepatoma cells, we detected the cell cycle after knocking down ARHGEF11. Increased G0/G1 phase and decreased S phase proportions were observed in ARHGEF11-silenced hepatoma cells (Figure 2C). In addition, downregulation of ARHGEF11 induced apoptosis (Figure 2D). Moreover, it was shown that downregulation of ARHGEF11 significantly weakened the motilities of hepatoma cells (Figure 3A–3D). While, knockdown of ARHGEF11 had no impact on the cell viability and apoptosis of the non-neoplastic cell line L0-2, indicating the nonlethal character of ARHGEF11 (Supplementary Figure 1A and 1B). Collectively, these results demonstrated that ARHGEF11 functions as an oncogenic driver in cell growth and metastasis of HCC.

### ARHGEF11 modulates multiple signaling pathways implicated in cell proliferation and cell migration

**Figure 4:**
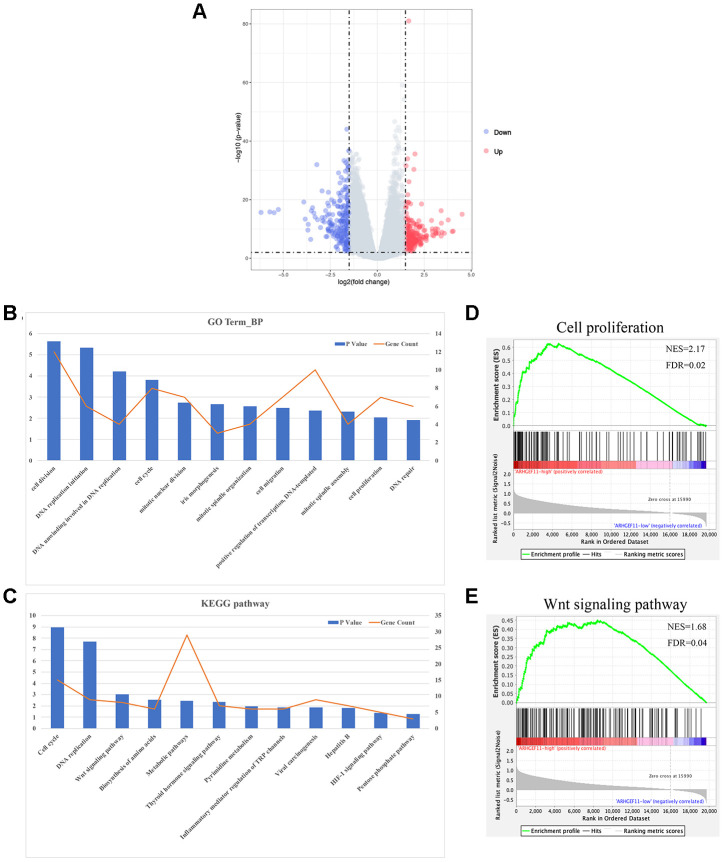
**Functional annotation and pathway enrichment analysis for ARHGEF11.** (**A**) Volcano plot of DEGs between ARHGEF11-high and ARHGEF11-low patients. (**B**) GO analysis of biological process (BP) using DAVID. (**C**) KEGG pathway analysis using DAVID. (**D** and **E**) GSEA analysis as a validation for the DAVID analysis.

To explore the molecular mechanisms of ARHGEF11-mediated HCC progression, we performed a bioinformatic analysis to profile the differentially expressed genes (DEGs) between ARHGEF11-high expression and ARHGEF11-low expression groups based on the RNAseq data from TCGA. A total of 458 DEGs were identified (|logFC|≥1.5, *P*<0.01), including 230 up-regulated genes and 228 down-regulated genes in ARHGEF11-high group compared to ARHGEF11-low group (Figure 4A). The GO and KEGG analysis showed that high expression of ARHGEF11 was related to cell proliferation, cell cycle, cell migration and Wnt signaling pathway (Figure 4B and 4C). Moreover, GSEA analysis revealed that high ARHGEF11 expression was associated with the functional gene sets of cell proliferation and Wnt signaling pathway (Figure 4D and 4E). Given the fact that Wnt/β-catenin signaling pathway was closely related to both cell proliferation and cell migration processes [9, 15], these results implied that ARHGEF11 might regulate HCC cell proliferation and cell migration via Wnt/β-catenin signaling pathway.

### The induction of EMT, cell cycle progression and cell apoptosis inhibition by ARHGEF11 is associated with β-catenin nuclear translocation

**Figure 5:**
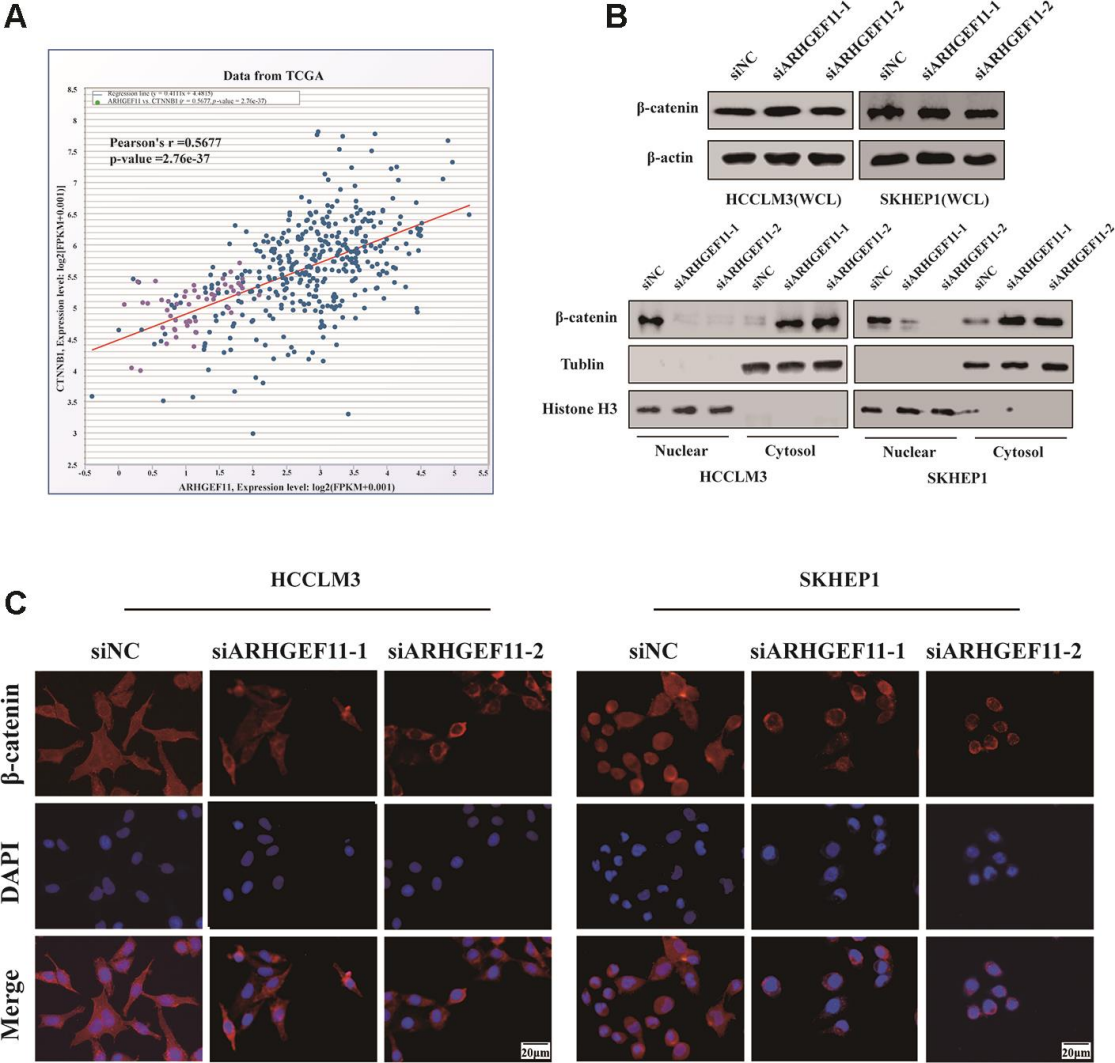
**Knockdown of ARHGEF11 attenuates EMT, cell cycle progression and induces cell apoptosis via β-catenin-dependent way.** (**A**) Correlation between the β-catenin and ARHGEF11 expression from TCGA HCC dataset analyzed by online CHIPbase. (**B**) β-Catenin expression from whole-cell lysates (up panel), the cytoplasmic and cell nuclear lysates (below panel) detected by western blot. (**C**) β-Catenin expression determined via immunofluorescence.

To verify whether ARHGEF11 could regulate Wnt/β-catenin signaling pathway, we first tested the correlation between the mRNA expression levels of ARHGEF11 and β-catenin in TCGA dataset. The results showed a positive correlation between ARHGEF11 and β-catenin expression (Figure 5A). Next, we examined the protein expression levels of β-catenin after deleting ARHGEF11. As shown in Figure 5B, although the total β-catenin expression remained unchanged, we found that knockdown of ARHGEF11 induced an increase of cytolytic β-catenin levels and a decrease of nuclear β-catenin levels (Figure 5B). Additionally, immunofluorescence assay was performed to further explore the sub-cellular localization of β-catenin. Similarly, it was shown that β-catenin primarily accumulated in the cell nuclear of ARHGEF11-siNC cells, however, downregulation of ARHGEF11 significantly attenuated nuclear translocation of β-catenin, most of which were detained in the cytoplasm (Figure 5C). These results suggested that ARHGEF11 could activate β-catenin pathway through inducing nuclear translocation of β-catenin.

**Figure 6:**
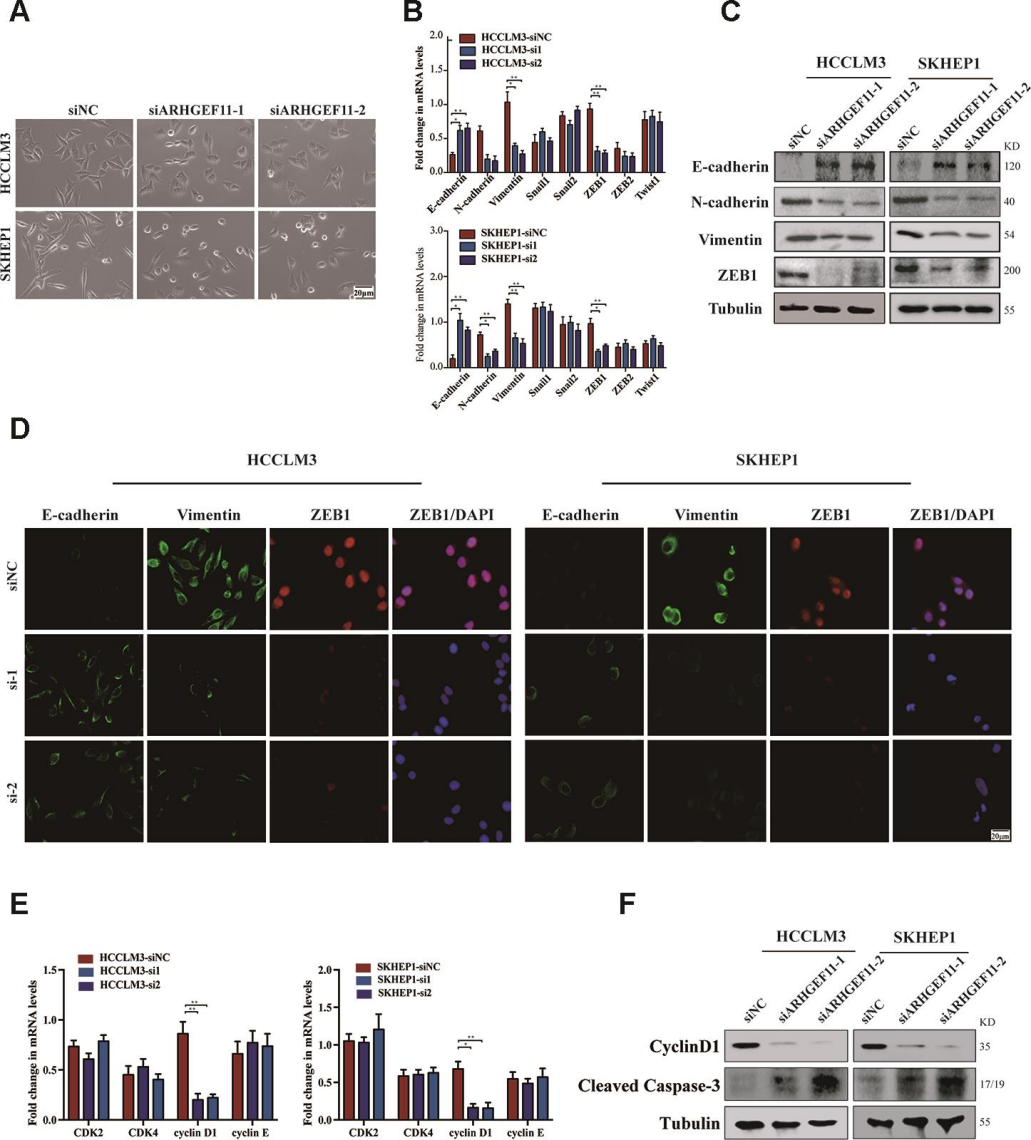
**Knockdown of ARHGEF11 attenuates EMT, cell cycle progression and induces cell apoptosis via β-catenin-dependent way.** (**A**) Micrographs of indicated cell clones. (**B**) The mRNA expression of a series of EMT markers and inducers in indicated cells. (**C**) Relative expression of E-cadherin, N-cadherin, Vimentin and ZEB1 detected by western blot in the indicated cells. (**D**) Immunofluorescence assay of E-cadherin, Vimentin, and ZEB1 expression in the indicated cells. The merged images show overlays of ZEB1 (red) and nuclear staining by DAPI (blue); scalebar: 20 mm. (**E**) The mRNA levels of CDK1, CDK2, cyclinD1 and cyclinE in indicated cells (**F**) Relative expression of cyclinD1 and Cleaved Caspase-3 detected by western blot in the indicated cells.

Activation of β-catenin has been shown to induce cell migration via ZEB1 (an important transcription factor during EMT)-mediated EMT process [16]. We further investigated whether ARHGEF11-induced β-catenin nuclear translocation could stimulate EMT process through upregulation of ZEB1. Silencing ARHGEF11 of the hepatoma cells induced an epithelial phenotype (Figure 6A). The mRNA levels of E-cadherin, N-cadherin, Vimentin and a panel of EMT inducer were determined by qRT-PCR (Figure 6B). Knockdown of ARHGEF11 in HCCLM3 and SKHEP1 cell lines caused a decrease of ZEB1 and an increase of E-cadherin, an epithelial cell marker that was negatively regulated by ZEB1 [17]. Consistently, N-cadherin and Vimentin, two mesenchymal cell makers, were upregulated by downregulation of ARHGEF11 (Figure 6B). However, ARHGEF11 had no impact on the other EMT inducers. These results were also verified by western blot and immunofluorescence analysis. ARHGEF11-silenced hepatoma cells displayed a clear increase in E-cadherin and a decrease in Vimentin as well as ZEB1 compared with the control group (Figure 6C and 6D).

On the other hand, β-catenin has also been proved to regulate cell proliferation via upregulation of cyclinD1 [18]. As a target gene of β-catenin, cyclinD1 displayed a lower level after ARHGEF11 downregulation, suggesting an arrested state of cell cycle (Figure 6E and 6F). Moreover, knockdown of ARHGEF11 enhanced the expression of Cleaved Caspase-3, indicating a vital role of ARHGEF11 in regulating cell apoptosis process (Figure 6F). To further confirmed the role of β-catenin in the ARHGEF11-induced HCC progression. We used the β-catenin agonist LiCl (40 mM), which could completely rescue the phenotype of ARHGEF11 silence, including proliferation and migration of hepatoma cells (Supplementary Figure 2A, 2B). Taken together, these results implied that ARHGEF11 upregulation might lead to β-catenin nuclear translocation, thereby inducing EMT, cell cycle progression and cell apoptosis inhibition.

### Upregulation of ARHGEF11 in human HCC patients predicted poor survival outcomes

**Figure 7:**
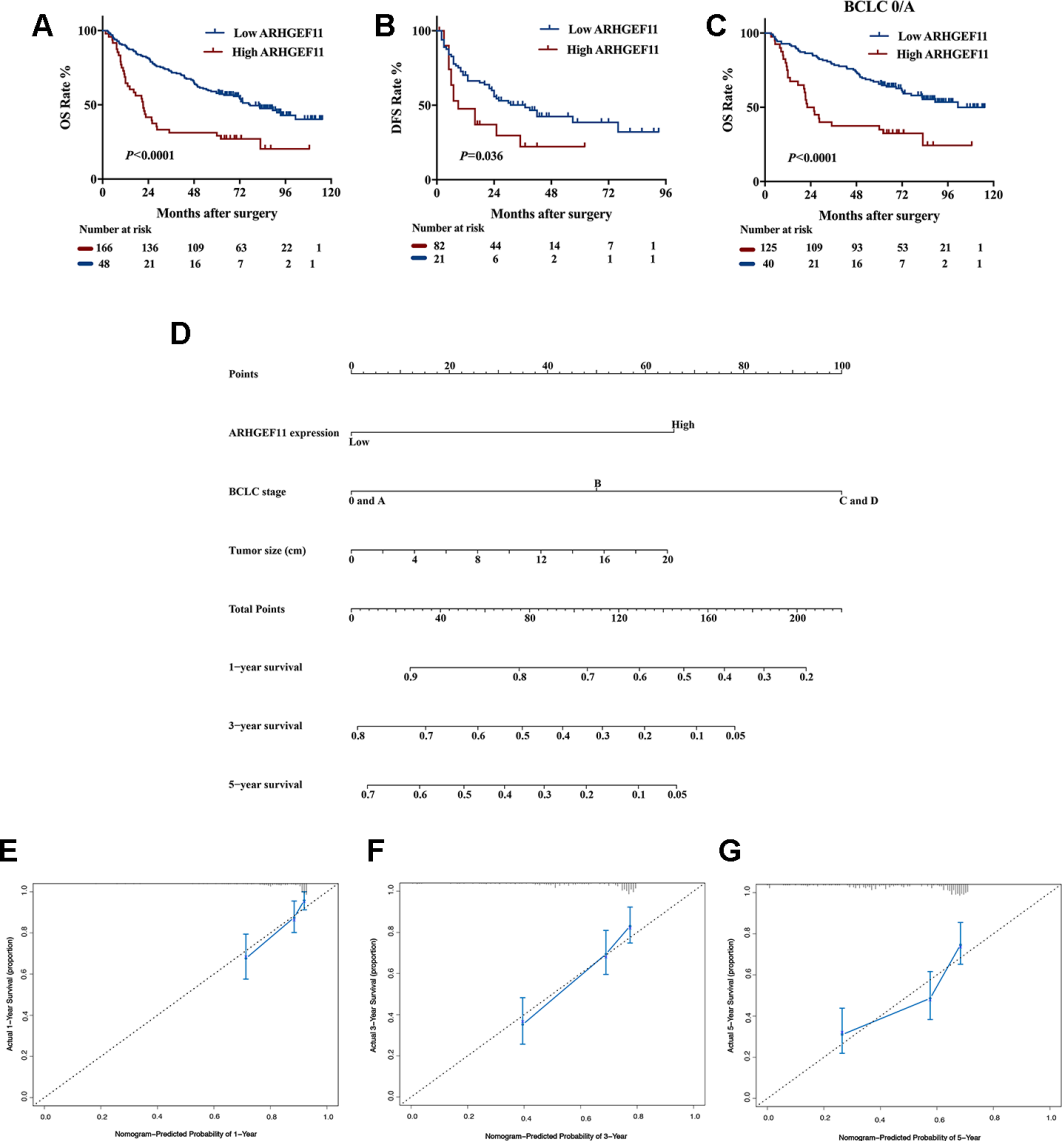
**Upregulation of ARHGEF11 in human HCC patients predicted poor survival outcomes.** Kaplan-Meier curve about OS (**A**) and DFS (**B**) of patients from West China hospital dataset based on ARHGEF11 high and ARHGEF11 low expression. (**C**) Kaplan-Meier curve about OS of patients with BCLC 0/A classification from West China hospital dataset based on ARHGEF11 high and ARHGEF11 low expression. (**D**) Nomogram for OS that integrated ARHGEF11 expression levels, tumor size and BCLC classification. (**E**–**G**) Calibration curve for predicting patients’ survival at 1 year, 3 year and 5 year respectively.

To reveal the clinical significance of ARHGEF11 overexpression, we divided a total of 214 HCC patients from West China Hospital dataset into ARHGEF11-high (n=166) and ARHGEF11-low (n=48) expression subgroups, according to the best cut-off point of ARHGEF11 expression analyzed by X-tile software. The clinicopathologic analysis indicated that higher ARHGEF11 expression was positively associated with advanced BCLC classification (Table 1). Kaplan-Meier analysis demonstrated that patients with higher ARHGEF11 levels had poorer OS and DFS outcomes (Figure 7A and 7B). In addition, patients with BCLC 0/A stage, an early stage of BCLC-staging classification, also displayed similar results (Figure 7C). Furthermore, univariate and multivariate analysis identified high ARHGEF11 expression, together with larger tumor size and advanced BCLC stage, as three independent risk factors for OS (Table 2). These independent risk factors were used to construct an OS risk estimation nomogram (Figure 7D). The discrimination performance for prognosis of HCC was internally validated through bootstrap method. This nomogram model displayed good accuracy in estimating overall survival rate, with a C-index of 0.70 (95% CI, 0.65 to 0.74). Furthermore, calibration curve demonstrated great accordance to predict 1-year and 3-year survival rate, while poor agreement on estimating 5-year survival rate (Figure 7E–7G). Taken together, these results indicated that ARHGEF11 could be used as a prognostic biomarker for HCC patients.

**Table 1:** Clinicopathologic characteristics of patients in the ARHGEF11-low and ARHGEF11-high group

**Patient Characteristics**	**ARHGEF11 low (N=166)**	**ARHGEF11 high (N=48)**	**P value**
Age			0.269
Median (range)	51 (18-79)	46.5 (22-75)	
Gender			0.399
(Male/Female)	140/26	38/10	
HBV			0.898
(Positive / negative)	143/23	41/7	
Tumor size (cm)	5.78±3.37	6.44±3.99	0.254
AFP			0.864
≤400 ng/mL	98	29	
>400 ng/mL	68	19	
AJCC-TNM stage			0.244
(I/II/III/IV)	78/46/36/6	22/14/7/5	
BCLC Classification			0.012
(0, A/B/C, D)	125/35/6	40/3/5	
Tumor number (Solitary/Multiple)	129/37	43/5	0.054
Cirrhosis (Yes/No)	105/61	30/18	0.526
ALT (U/L)	46.60±43.15	38.31±40.28	0.424
AST (U/L)	47.51±36.70	39.82±38.78	0.934

HBV: hepatitis B virus; AFP: alpha fetoprotein; ALT: alanine aminotransferase; AST: aspartate aminotransferase; AJCC: American Joint Committee on Cancer; BCLC: Barcelona Clinic Liver Cancer

**Table 2:** Univariate and multivariate analysis of risk factors associated with OS.

**Variable**	**Univariate analysis of risk factors associated with OS**			**Multivariate analysis of risk factors associated with OS**		
	**P**	**HR**	**95% CI**	**P**	**HR**	**95% CI**
Age	0.375	0.993	0.979-1.008	NA		
Gender (M *v* F)	0.120	0.658	0.389-1.114	0.135		
ARHGEF11	<0.001	2.465	1.660-3.661	**<0.001**	**2.861**	**1.878-4.359**
HBV (P *v* N)	0.387	1.270	0.739-2.180	NA		
Cirrhosis (Y *v* N)	0.249	1.247	0.858-1.813	0.082		
Tumor number (S *v* M)	0.001	1.917	1.284-2.861	0.222		
AFP	0.001	1.349	0.943-1.930	0.136		
Tumor size	0.002	1.075	1.027-1.124	**0.032**	**1.050**	**1.004-1.097**
AJCC-TNM stage	<0.001			0.056		
I						
II	0.001	2.125	1.386-3.258			
III	0.019	1.784	1.102-2.889			
IV	<0.001	5.458	2.708-11.001			
BCLC Classification	<0.001			**<0.001**		
0 and A						
B	0.001	2.040	1.341-3.105	**<0.001**	**2.516**	**1.615-3.920**
C and D	<0.001	4.368	2.238-8.524	**<0.001**	**3.910**	**1.983-7.711**

M: male; F: female; P: positive; N: negative; Y: yes; N: no; S: solitary; M: multiple; AFP: alpha fetoprotein; AJCC: American Joint Committee on Cancer; BCLC: Barcelona Clinic Liver Cancer

## DISCUSSION

ARHGEF11 is a member of RhoGEFs, which can catalyze Rho GTPases to hydrolyze GTP into GDP [19]. Rho GTPases has been demonstrated to be associated with many fundamental cellular functions, including cell cycle, vesicle trafficking and cytoskeleton assembly [20]. Given the crucial role of Rho GTPases in regulating cell cycle and cell motility, emerging evidences have demonstrated that RhoGEFs were implicated with cancer progression [21, 22]. For example, P115RhoGEF has been proved to promote cell proliferation of prostate cancer through regulation of extracellular Ca^2+^-induced choline kinase activation [23]. Likewise, upregulation of ARHGEF11 had been identified in gallbladder cancer, glioblastoma multiforme, breast cancer and colon cancer, and was related to tumor metastasis [24–26]. Overexpression of ARHGEF11 was required for TROY-induced glioblastoma tumor cell invasion and survival [12]. In addition, ARHGEF11 was shown to promote breast cancer cells motility via inhibiting cell tight junctions [14]. However, the functions of ARHGEF11 in HCC have yet to be determined. In our study, it was shown that ARHGEF11 promoted proliferation and EMT of hepatoma cells via activating β-catenin pathway. Moreover, the weakly metastatic cell lines (SNU-182, Hep3B and Huh7) expressed a lower level of ARHGEF11, suggesting that ARHGEF11 might be associated with the metastatic potential of hepatoma cells. Thus, our future work will focus on the point that whether ARHGEF11 is a key player in developing the metastatic characters in non-metastatic HCC. Equally important, the mechanism of ARHGEFF11 upregulation in HCC is unclear up to now. More works are needed to explore this issue in the future. In addition, the upregulation of ARHGEF11 in HCC tissues was closely related to poor survival outcomes of HCC patients. These results will broaden the knowledge about the roles of RhoGEFs in cancers and facilitate the assessment of RhoGEFs as prognostic factors or drug targets of cancers.

As a key factor of the canonical Wnt signaling pathway, β-catenin has been widely reported to be upregulated in HCC samples and promote hepatocarcinogenesis and metastasis [16, 27]. In the absence of Wnt signaling, β-catenin combines to E-cadherin to form a cytoplasmic complex locating at cell-cell junction. When this signaling pathway was activated by growth factors, TGFβ for instance, β-catenin detached from E-cadherin and translocated into the nucleus to regulate the transcription of a series of target genes, including ZEB1 and cyclinD1 [15]. Consistent with these facts, we showed that inhibition of ARHGEF11 did not affect total β-catenin expression, but repressed β-catenin nuclear translocation, which induced EMT suppression and cell cycle arrest via ZEB1 and cyclinD1. Furthermore, ARHGEF11 contains PDZ, a highly conserved domain also shared by dishevelled protein family and PDLIM family. Interestingly, both dishevelled protein and PDLIM1 (a member of the PDLIM family) have been reported to protect β-catenin from degradation through protein-protein interaction via PDZ domain, thereby enhance the nuclear translocation of β-catenin [28, 29]. Moreover, locating at cytoplasmic tight junction, ARHGEF11 depletion disrupted the formation of tight junction and peri-junctional actomyosin ring [30]. Taken together, these findings suggest that ARHGEF11 might induce β-catenin nuclear translocation via protein-protein interaction. However, this hypothesis requires further validation.

The clinical outcomes of HCC vary significantly among individuals because of the heterogeneity, even with the same TNM stage [31]. Accurate prognostic evaluation is crucial for stratification of patients, medical management by clinicians, as well as analysis of clinical trials. In this study, the ARHGEF11 expression displayed a potential to be used for clinical prognosis. Bases on a cohort of 214 patients, we showed that high expression of ARHGEF11 in HCC was a risk factor of poor survival. Nomogram has been used to predict patient’s survival probability and is shown to be more accurate than the conventional staging system [32]. In this study, we constructed a single numerical estimated model of nomogram that integrated ARHGEF11, tumor size and BCLC classification. The proposed nomogram performed well as supported by the C index of 0.70 and the calibration curves demonstrated an optimal agreement between prediction and actual observation. As the molecular alterations of HCC may determine whether malignant events proceed or not, applying various biomarkers to estimate the survival outcomes is a main strategy in exploring clinical predictors. Our study demonstrated the clinical relevance and prognostic significance of ARHGEF11, which may provide a potential candidate of biomarker for HCC.

**Figure 8:**
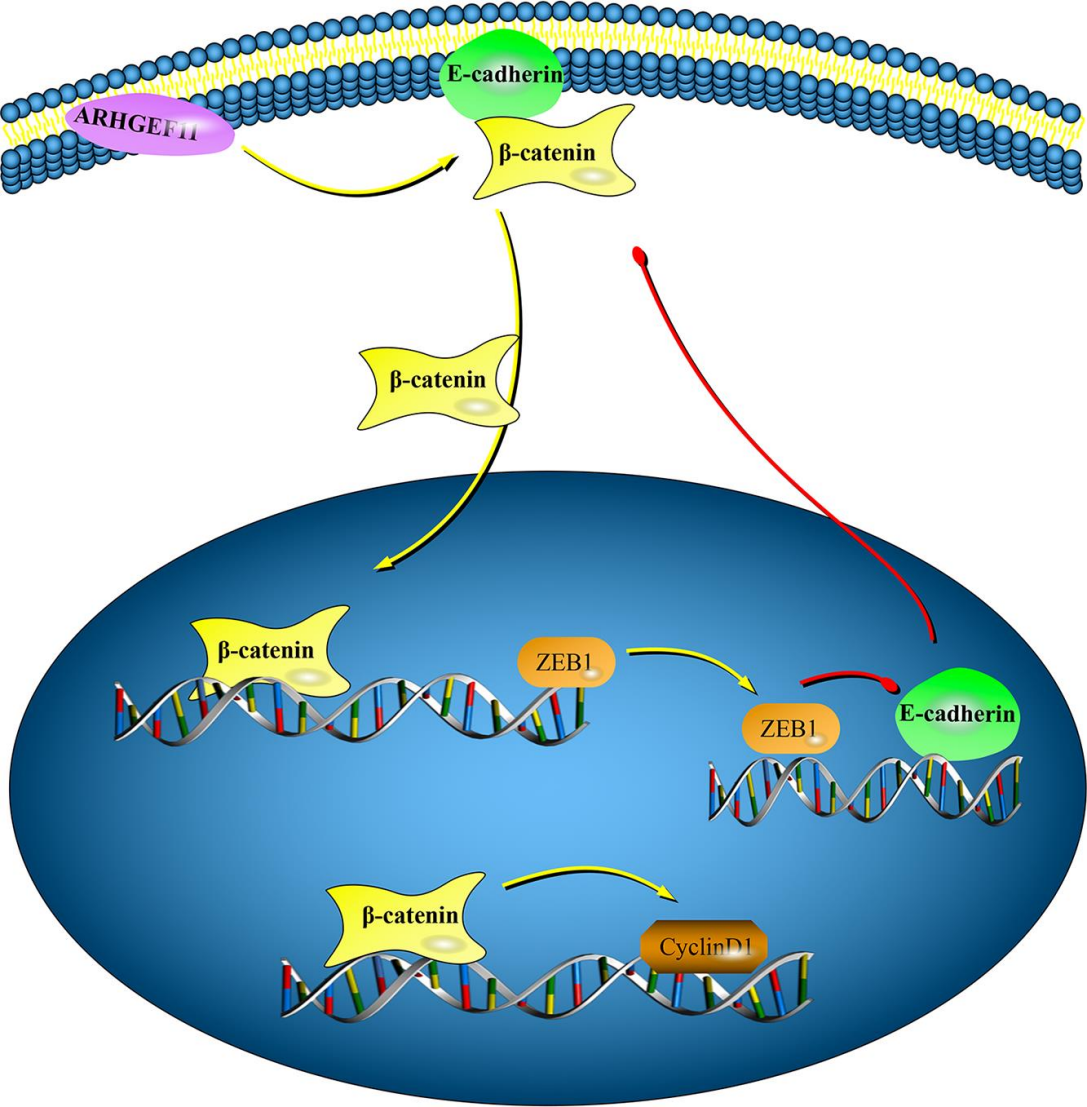
**Graphical model displaying the mechanisms of ARHGEF11 in regulating cell cycle and EMT via activating β-catenin.** Upregulation of ARHGEF11 promotes β-catenin nuclear translocation from cell membrane. β-catenin activates cyclinD1 transcription, which then leads to cell cycle progression. At the same time, β-catenin enhances ZEB1 transcription, which can inhibit E-cadherin expression, thereby inducing EMT.

In conclusion, this study revealed a new function of ARHGEF11 in regulating cell cycle and EMT through a β-catenin dependent pathway in HCC (Figure 8). High expression of ARHGEF11 were correlated with worse prognosis of HCC patients, Therefore, ARHGEF11 could be used as a potential biomarker of HCC.

## MATERIALS AND METHODS

### Patients and follow-up

All HCC tissues and corresponding para-tumor tissues were obtained from patients who had undergo surgical resections between 2007-2012 at West China Hospital (Chengdu, China). Patients’ data including gender, age, HBV infection status, TNM stage, BCLC classification, tumor number, tumor size, liver cirrhosis, and preoperative serum AFP, AST, ALT levels were also collected. The follow-up information was acquired by clinic visits or telephone interviews. Disease free survival (DFS) was defined as the time of surgery to clinical evidence of tumor recurrence or metastasis. Overall survival (OS) was calculated from the period of operation to the time of patient death or last follow-up. This study was approved by the Ethical Review Committees of Sichuan University (Chengdu, China) and the written informed consents were collected from all patients.

### Immunohistochemistry (IHC)

The process of immunohistochemical staining was operated as previous report [33]. Briefly, the paraffin sections were heated at 60 ° C for 60 minutes, then dewaxed to xylene twice for 15 minutes and washed by gradient alcohol and distilled water for 5 minutes. The slides were placed in boiling citrate buffer (pH = 9.0) for 10 minutes, followed by incubating with 3% H_2_O_2_ to block peroxidase. After washed with PBS, the slides were incubated with the primary antibodies overnight at 4°C, followed by incubation with secondary antibody for 30 minutes and DAB solution for chromogenic reaction. Two experienced pathologists who were blended to this study performed the evaluation of the results. The assessment of ARHGEF11 expression was performed as following: 5 respective views at 400× magnification from each slide were recorded. The quantification of ARHGEF11expression is performed according to the intensity of the stained tissues and the percentage of positive cells. The maximum score is up to 300. The mean score from five views was for further analysis.

### Quantitative real-time polymerase chain reaction (qRT-PCR)

Total RNA from cells was isolated using RNAiso Plus kit (Takara) and was reversely transcribed to cNDA using PrimeScript™ RT Master Mix reagent (Takara), followed by PCR amplifications with SYBR® Premix Ex Taq™ (Takara). The relative mRNA levels were normalized to GAPDH and analyzed by using the 2^-ΔΔCT^ method in this study. The specific primer sequences were listed in Supplementary Table 1.

### Western blot

Total protein was extracted using cold RIPA buffer with cocktail and quantified by BCA methods (Beyotime Biotechnology, China). Nuclear and cytoplasmic protein extraction was performed using commercial Nuclear and Cytoplasmic Extraction Reagents (AM1921, Thermo Scientific). Protein samples were denatured and separated by electrophoresis on 10% polyacrylamide gels, followed by transferred onto PVDF membranes. The membranes were blocked with 5% skim milk powder for 1 hour at room temperature. After incubating with various primary antibodies (4° C, overnight) and corresponding secondary antibodies (room temperature, 1 hour), blot band was examined with enhanced chemiluminescence (ECL) reagent and analyzed with Image Lab software. Antibodies used in this study were as follows: ARHGEF11 (Santa Cruz, sc-166740), Vimentin (Santa Cruz, sc-6260), N-cadherin (Santa Cruz, sc-393933), E-cadherin (Santa Cruz, sc-71008), ZEB1 (Santa Cruz, sc-515797), β**-**tubulin (Santa Cruz, sc-166729), β**-**actin (Santa Cruz, sc-47778), Histone H3 (CST, 4499), CyclinD1 (CST, 2922), Cleaved Caspase-3 (CST, 9661), β-catenin (CST, 8480).

### Prediction of ARHGEF11 function using bioinformation analysis

Raw data of gene expression were obtained from TCGA and then were transformed into RPKM (Reads Per Kilobase per Million mapped reads) for the differential expression genes (DEGs) analysis. Statistical ranking of ARHGEF11 expression in the top quartiles was defined as high ARHGEF11 expression group, while the bottom quartiles were low ARHGEF11 expression group. The edgeR package in RStudio version 1.2.5001 was used to calculated DEGs between high and low ARHGEF11 expression groups. Genes with adjust *P* value < 0.01 and absolute log2 (fold change) ≥1.5 were considered statistically significant. DEGs were used to perform Gene Ontology (GO) analysis using web-accessible program DAVID as well as Gene set enrichment analysis (GSEA) reported by other studies [34].

### Cell lines

SNU-182, Huh-7, Hep3B and SKHEP1 cell lines were purchased from Cell Bank Type Culture Collection Committee (CBTCCC, Shanghai, China). HCCLM3 cell line was obtained from State Key Laboratory Biotherapy, West China Hospital (Chengdu, China). All cells were cultured in 5% CO_2_ at 37 °C and incubated in DMEM (HyClone, Logan, USA) containing 10% FBS (HyClone).

### Transfection

Two small interfering RNA targeting ARHGEF11 (siARHGEF11-1, siARHGEF11-2) and a negative controls (siNC) were provided by Viewsolid Biotech (Beijing, China). After 80% confluence, HCCLM3 and SKHEP1 cells were transfected with corresponding siRNA by using GenMute transfecting reagent (SignaGen) according to the manufacture instructions. After 48h, the ARHGEF11 protein expression was determined by immunoblotting. The siRNA sequences were as follow: siARHGEF11-1: 5’-GGUGUAGAUCAAAGCCCAATT-3’; siARHGEF11-2:5’- GCCGGGAG AUC UCA AGUATT-3’.

### CCK8 assay for cell viability

A tall of 2000 cells in a volume of 100μl medium per well were planted in 96-well plates with five replicates for each sample. At 0h, 24h, 48h, 72h, 96h and 120h, the CCK-8 reagent (10μl) was added into each well and incubated for 2h at 37 °C. The absorbance was detected at 450nm by ELISA reader.

### Cell apoptosis and cell cycle assay

To evaluate the cell apoptosis, cell suspension was washed with phosphate buffered saline (PBS), and mixed with binding buffer. The mixture was then cultured with Annexin V/FITC and PI (4A Biotech, China) for 5 minutes at room temperature, followed by Cytoflex Flow Cytometer (Beckman, USA) detecting. For cell cycle assay, cells at log-phase growth were collected, washed in cold PBS, and fixed with 95% cold ethanol for overnight. After that, cells were incubated with 7-AAD buffer (4A Biotech, China) for 30 minutes at dark room, then determined by Flow Cytometer.

### Wound healing, cell migration and invasion assay

For the wound healing assay, cell suspension was added into 12-well plates. After 80% cell confluence, a homogenous wound was made with a micropipette tip. The cell wounded area was photographed at 0h, 24h, and 48h after wound scratching. For transwell migration and invasion assay, 5×10^4^ cells in serum-free medium were added into the upper transwell chamber with pore size 8 μm (Corning, China), which was coated with (invasion) or without (migration) Matrigel (BD, China), while the lower chamber was filled with migration-inducing medium (30% FBS). After 24h incubation, the cells on the bottom membrane were fixed with 4% paraformaldehyde and then stained with 0.1% crystal violet, while the cells on the upper membrane were removed with a cotton swab. After washed by PBS, the chambers were photographed via microscope.

### Immunofluorescence assay

Transfected cells seeded on coverslips in a 24-well plate. After 24h, the coverslips were washed with PBS, fixed with 4% paraformaldehyde and permeabilized with 0.2% Triton X-100 buffer for 10 minutes at room temperature. The cells were next blocked with 5% BSA for 2 hours, and then were incubated with primary antibody (diluted 1:100) overnight at 4 °C, followed by incubation with appropriate fluorophore-conjugated secondary antibody (Thermo Scientific, USA) for 2 hours at 37 °C and DAPI (4A Biotech, China) for 10 minutes at room temperature. Images were captured by aAX10 imager A2 microscope (Carl Zeiss MicroImaging), and processed using the software provided by the manufacturer.

### Statistical analysis

All data analysis was performed using SPSS 23.0 and Prism version 6.0 (Graphpad) software. For continuous variables, data was shown as mean ± standard deviation (SD). Unaired or paired t test was used to compared to two groups. The relationships between ARHGEF11 expression and clinicopathological features of HCC patients were analyzed by *x^2^* test or Fisher’s exact test, while the correlation between ARHGEF11 and β-catenin expression was performed by Pearson’s correlation analysis (Online CHIPbase: http://rna.sysu.edu.cn/chipbase/index.php). Survival rate was calculated using Kaplan-Meier method and compared by the Log-rank test. Cox regression analysis was used to perform univariate and multivariate analysis for OS.

The nomogram, based on the results multivariate analysis, was constructed by using the package of *rms* in RStudio. For nomogram validation, concordance index (C-index) and Calibration plot, both using 1,000 bootstrap resamples method, were introduced. C-index, which indicated the discrimination ability of the nomogram, was positively correlated with the accuracy of the prognostic prediction. While the calibration plot measures how closely the nomogram-predicted probabilities agree with the actual observed survival probability. *P*<0.05 was considered statistically significant and all statistical tests were two-sided.

## Supplementary Material

Supplementary Figures

Supplementary Table 1
