# Supplementary material for: ARHGEF11 promotes proliferation and epithelial-mesenchymal transition of hepatocellular carcinoma through activation of β-catenin pathway
Source: Aging (Albany NY). 2020 Oct 29;12(20):20235–53. doi: 10.18632/aging.103772 (PMC7655160; doi:10.18632/aging.103772)
Supplement: Supplementary Figures [file aging-12-103772-s001..pdf]

SUPPLEMENTARY FIGURES

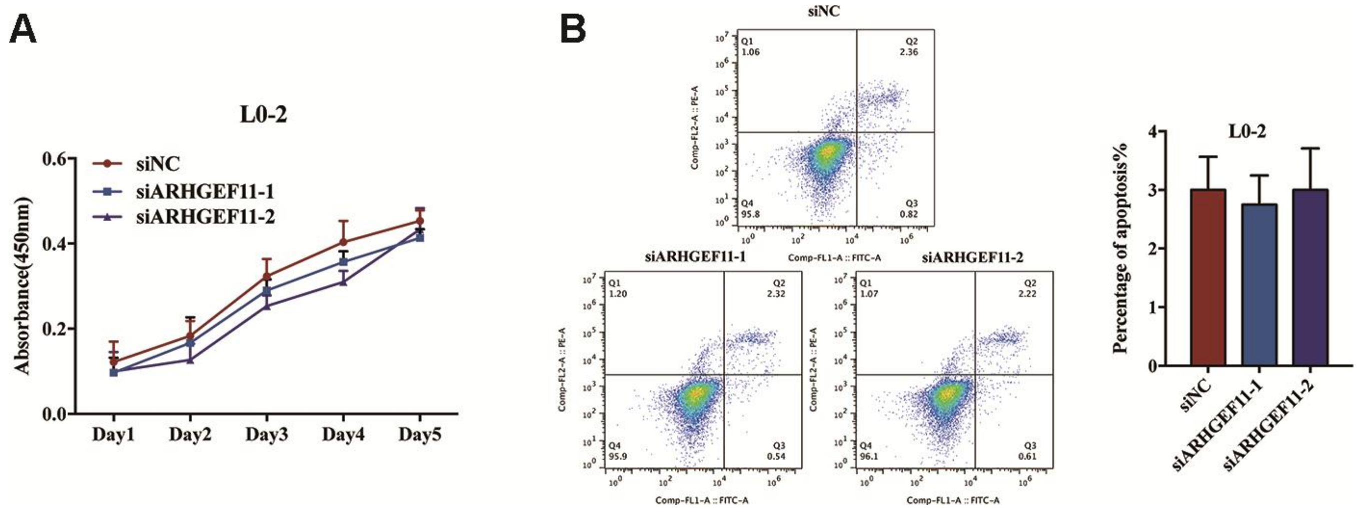

Supplementary Figure 1. Knockdown of ARHGEF11 had no impact on the cell viability and apoptosis of the non-neoplastic cell line L0-2. (**A**) The viability of hepatoma cells detected by CCK8 assay. (**B**) Cell apoptosis detected by flow cytometry.

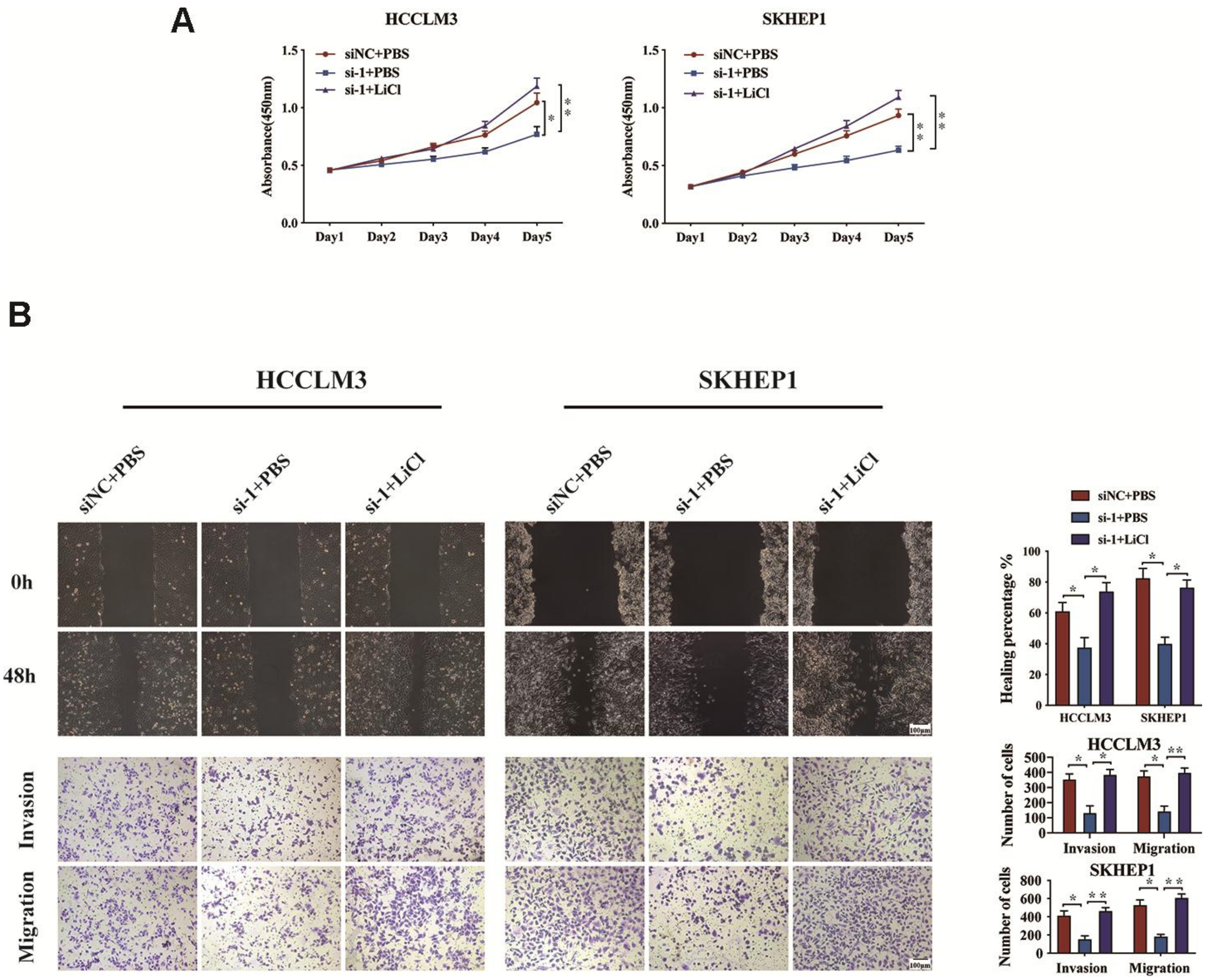

Supplementary Figure 2. The β-catenin agonist, LiCl (40 mM for 6 hours), could completely rescue the phenotype of ARHGEF11 silence. (**A**) The viability of hepatoma cells detected by CCK8 assay. (**B**) Migration ability examined by Wound healing assay (up panel). The migration and matrigel invasion ability detected by Transwell assay (below panel).
