# Supplementary material for: ARHGEF11 promotes proliferation and epithelial-mesenchymal transition of hepatocellular carcinoma through activation of β-catenin pathway
Source: Aging (Albany NY). 2020 Oct 29;12(20):20235–53. doi: 10.18632/aging.103772 (PMC7655160; doi:10.18632/aging.103772)
Supplement: Supplementary Table 1 [file aging-12-103772-s002..pdf]

SUPPLEMENTARY TABLE

Supplementary Table 1. The sequences of PCR primers.

Primer	Sequence 5'-3'
ARHGEF11 Forward	GGATCGCATTGTTCTGGTGC
ARHGEF11 Reverse	CCATGGTGCCGTTGACTTTG
GAPDH Forward	GGAGCGAGATCCCTCCAA AAT
GAPDH Reverse	GGCTGTTGTCATACTTCTCATGG
E-cadherin Forward	AGCCCCGCCTTATGATTCTCTG
E-cadherin Reverse	TGCCCCATTCGTTCAAGTAGTCAT
N-cadherin Forward	TTTGATGGAGGTCTCCTAACACC
N-cadherin Reverse	ACGTTTAACACGTTGGAAATGTG
Vimentin Forward	GACGCCATCAACACCGAGTT
Vimentin Reverse	CTTTGTCGTTGGTTAGCTGGT
Snail1 Forward	GACCCCAATCGGAAGCCTAACTAC
Snail1 Reverse	AGCCTTTCCCACTGTCCTCATC
Snail2 Forward	CCTCCATCTGACACCTCC
Snail2 Reverse	CCCAGGCTCACATATTCC
ZEB1 Forward	AAGTGGCGGTAGATGGTA
ZEB1 Reverse	TTGTAGCGACTGGATTTT
ZEB2 Forward	TTCTGCGACATAAATACG
ZEB2 Reverse	GAGTGAAGCCTTGAGTGC
Twist1 Forward	CTCAAGAGGTCGTGCCAATC
Twist1 Reverse	CCCAGTATTTTTATTTCTAAAGGTGTT
CDK1 Forward	AAACTACAGGTCAAGTGGTAGCC
CDK1 Reverse	TCCTGCATAAGCACATCCTGA
CDK2 Forward	CCAGGAGTTACTTCTATGCCTGA
CDK2 Reverse	TTCATCCAGGGGAGGTACAAC
cyclinD1 Forward	GCTGCGAAGTGGAAACCATC
cyclinD1 Reverse	CCTCCTTCTGCACACATTTGAA
cyclinE Forward	AAGGAGCGGGACACCATGA
cyclinE Reverse	ACGGTCACGTTTGCCTTCC
